# Gold nanoparticles: challenge of PPPM for heart failure treatment and drug delivery

**DOI:** 10.1186/1878-5085-5-S1-A90

**Published:** 2014-02-11

**Authors:** Mykola Ya Spivak, Rostyslav V Bubnov, Ilya M Yemets, Liudmyla M Lazarenko, Natalia O Tymoshok, Zoia R Ulberg

**Affiliations:** 1 Zabolotny Institute of Microbiology and Virology, National Academy of Sciences of Ukraine, Kyiv, Ukraine; 2 Scientific-Practical Centre of Pediatric Cardiology and Cardiac Health of Ukraine, Kyiv, Ukraine; 3 Ovcharenko Institute of Biocolloidal Chemistry, National Academy of Sciences of Ukraine, Kyiv, Ukraine

## Introduction

It was demonstrated the wide potential for biomedical applications of nanoscale gold particles because of their unique biological properties [1,2]. The use of gold nanoparticles in cardiology is promising to develop fundamentally new methods of diagnosis and treatment.

## Aims and objectives

The aim was to test effects of nanoparticles using suggested congestive heart failure rat model, applying intrapleural and intraveonous injection, to compare with proved medication Simdax; to test gold nanoparticle for drug delivery, to test sonoporation effect to increase nanoparticles delivery into myocardial cell in rats.

## Material and methods

Wistar rats weighing 180-200 g (n=54) of each sex were selected on the basis of analogies for experiment, within two weeks received intravenous injection of doxorubicin Sigma in cumulative dose of 12.0 mg / kg to model advance heart failure, registered by ultrasonography using ultrasound transducers up to 12 MHz. At 14^th^ day we formed 6 groups with 8 rats in each: first three groups of animals received simdax, nanoparticles and gold conjugate (simdax, nanogold) into pleural cavities in a dose of 0.06 ml per animal. 7th (n=8) group was control (saline). The fourth, fifth and sixth groups of animals received simdax, gold nanoparticles and conjugate intravenously in same dose. We considered hydrothorax as most representative sign for effective dynamic assessment of heart failure regression. Sonoporation of gold nanoparticles to cardiomyocytes was tested.

## Results

In all animals of 6 groups after 3rd day after medication injection no ascites, no liver enlargement was registered (compare to controls p<0.001). The linear cranio-caudal measurements of fluid level pleural cvities were as followed: for gold nanoparticles intrapleural right 1.8 ±0.11 mm; left – 2.1±0.13 mm, compared to hydrothorax in controls right 6 ±0.46 mm; left 7 ±0.35 mm. Conjugate injection shown significantly higher hydrothorax reduction than injection of Simdax only (p<0.01); gold nanoparticles injection shown significantly higher than injection of Simdax (P<0.05) gold nanoparticles and conjugate shown non significant difference in rat recovery. Difference in rat life continuity was significant between simdax vs. nanogold P < 0.05) and simdax vs conjugate (P < 0.05). Sonoporation enhances AuNPs transfer into cell and mytochondrias that were highly localized, and was superior to controls (P <0.01 for both).

## Conclusions

Gold nanoparticles conjugated with cardiotropic drugs have significant cardioprotective effects for doxorubicin induced heart failure rats, higher than Simdax only. Gold nanoparticle alone show effects similar to the conjugate injection. Intrapleural (local delivery) route is preferred over intravenous (systemic) according to all tested parameters. Sonoporation is able to enhance gold nanoparticle delivery to myocardial cells in vivo.

## Outlook and expert recommendations

It is recommended to create international project to study gold nanoparticles for development nanoconstructions to treat patients with heart failure. Extend studies to nanoparticles application for neurodegenerative, heart, liver, kidney diseases, muscle dystrophy, combining with biological therapies to archive sustainable effects from theranostic approach.
